# A computational multi-targeting approach for drug repositioning for psoriasis treatment

**DOI:** 10.1186/s12906-021-03359-2

**Published:** 2021-07-05

**Authors:** Akachukwu Ibezim, Emmanuel Onah, Ebubechukwu N. Dim, Fidele Ntie-Kang

**Affiliations:** 1 Department of Pharmaceutical and Medicinal Chemistry, University of Nigeria, Nsukka, Nigeria; 2 Department of Science Laboratory and Technology, University of Nigeria, Nsukka, Nigeria; 3 Department of Chemistry, University of Buea, Buea, Cameroon; 4 Department of Pharmaceutical Chemistry, Martin-Luther University Halle-Wittenberg, Halle (Saale), Germany

**Keywords:** Binding free energies, Docking, Drug repositioning, Psoriasis

## Abstract

**Background:**

Psoriasis is an autoimmune inflammatory skin disease that affects 0.5–3% of the world’s population and current treatment options are posed with limitations. The reduced risk of failure in clinical trials for repositioned drug candidates and the time and cost-effectiveness has popularized drug reposition and computational methods in the drug research community.

**Results:**

The current study attempts to reposition approved drugs for the treatment of psoriasis by docking about 2000 approved drug molecules against fifteen selected and validated anti-psoriatic targets. The docking results showed that a good number of the dataset interacted favorably with the targets as most of them had − 11.00 to − 10.00 kcal/mol binding free energies across the targets. The percentage of the dataset with binding affinity higher than the co-crystallized ligands ranged from 34.76% (JAK-3) to 0.73% (Rac-1). It was observed that 12 out of the 0.73% outperformed all the co-crystallized ligands across the 15 studied proteins. All the 12 drugs identified are currently indicated as either antiviral or anticancer drugs and are of purine and pyrimidine nuclei. This is not surprising given that there is similarity in the mechanism of the mentioned diseases.

**Conclusion:**

This study, therefore, suggests that; antiviral and anticancer drugs could have anti-psoriatic effects, and molecules with purine and pyrimidine structural architecture are likely templates to consider in developing anti-psoriatic agents.

## Background

Psoriasis is an autoimmune inflammatory skin disease that affects 0.5–3% of the world’s population [1]. It is caused by the complex interplay of the innate and adaptive immune systems together with a wide array of genetic and environmental factors. Environmental triggers such as stress, injury, drugs, and the disease start the self-propelled cycle of inflammation culminating in hyper-proliferation due to the activation of the innate immune system cells [2–5]. The disease is associated with decreased quality of life and multiple comorbid conditions, including metabolic syndrome, cardiovascular diseases, obesity, diabetes type 2 and Crohn’s disease [6–8]. In spite of effort by many researchers, there is currently no drug for curing the disease only for management purposes. Therefore, chemotherapy for this disease is highly needed [9, 10].

The goal of drug repurposing is to discover new uses of old (known) drugs [11]. The field of drug repositioning is growing rapidly because it starts from compounds, which are often Food and Drug Administration (FDA) approved drugs, with well-characterized pharmacology and safety profiles. Benefits accompanying this strategy are reduction in the risk of attrition in drug development during clinical trials and subsequently cost [12, 13]. Moreover, the successes recorded through this strategy have popularized its use in drug discovery courses. For example, Pfizer’s sildenafil is now also prescribed for erectile dysfunction, Celgene’s thalidomide is repurposed for cancer, Upjohn’s minoxidil for alopecia and so on [14, 15]. When compared with diseases like cancer where previously known drugs have been repurposed, e.g. the painkiller aspirin for cancer prevention, metformin previously known for the treatment of type-2 diabetes to protect against cancer development [16, 17], no repurposed drug has been established as a treatment for psoriasis to date, thus suggesting the current study.

The use of computers and computer software in drug research has become a common practice because it saves cost and time. In addition, due to advances in technology and computer power, accuracy of theoretical results is improving significantly and as such their predictions represent experimental results more and more [18–23]. In our previous investigation of the medicinal plant, *Psorospermum febrifugum* Spach, we provided evidence that confirm its ethnopharmacological usage as antipsoriatic agent and further identified forty-two fatty acids from the GC-MS chromatogram of the most active extract fraction which could be responsible for its biological activity [24].

In this study, efforts have been made to screen about 2000 currently approved drugs against fifteen selected and validated anti-psoriatic drug targets through molecular docking with the aim of repurposing strong binders for the treatment of the disease. Analysis to identify drugs with better docking scores than co-crystallized ligands across the entire protein targets was made and their common motif noted. The binding modes of the most interesting candidate in the proteins’ cavities were finally examined.

## Materials and methods

### Preparation of the approved drugs for molecular docking

The coordinate files, numbering 1852, of the approved drugs were retrieved from the ZINC database [25] and prepared by the Molecular Operating Environment software (MOE) [26]. The MOE 3-dimensional (3-D) protonation tool and MMFF94 force field [27] were, respectively, used to protonate the structures of the dataset and generate low energy structures to a gradient of 0.001 kcal/mol at 300 K and pH of 7.0.

### Preparation of the target proteins for modeling purpose

Fifteen selected X-ray crystal structures of enzymes implicated in the disease mechanism alongside their co-crystallized ligands were retrieved from the protein data bank [28]. Water and other non-essential small molecules co-crystallized with the protein-ligand complexes were deleted and then polar hydrogen atoms were added after which their low energies were generated using the ffG53a6 in Gromacs 4.5.5 [29]. Finally, each protein and their co-crystallized ligand was separated and saved as separate files.

### Docking procedure

The cavities occupied by each co-crystallized ligand were considered as the binding site of the fifteen anti-psoriatic targets and three main stages implemented in MOE DockTool were employed in docking the dataset into them as follows: First the program performed a systematic search to generate all combinations of angles for each ligand from its single 3-D conformation. Next Triangle Matcher tool placed a collection of poses, generated from the pool of ligand conformations, into the protein target binding site. Finally, London dG scoring function computed the binding free energy of the ligand from a given pose by taking cognizance of plethora of factors such as the average rotational and translational entropy terms, energy lost as a result of the flexibility of the ligand, hydrogen bonding, metal contacts and a desolvation term due the volumes of the atoms of the protein and ligand in contact with solvent. The program was set to retain the top 5 poses for each ligand. Note that the docking parameters were validated by using only the 3-D affinity grids which reproduced the experimental poses of the co-crystallized ligands within root mean square deviation (rmsd) of < 2.0 Å.

## Results and discussion

Since psoriasis is a chronic disease with no known cure, available drugs are only used to manage the symptoms and improve patients’ quality of life [30]. Thus, drugs for the management of psoriasis should be safe for long term use. They should also be cost-effective and very convenient to administer. However, the current drugs for the management of psoriasis have limitations ranging from lack of potency (topical agents), high toxicity (anticancer agents), high cost and relatively large molecular size. Furthermore, biologics require engineering from live, specialized cells [31, 32]. Due to these limitations, these drugs are not readily accessible to patients and those that can afford them still have problems with compliance because of the high untoward effects. This situation provided the impetus to search for better, cost-effective and safer anti-psoriatic drugs.

### Virtual screening of the approved drugs on the selected Antipsoriatic targets

Several parameters for the centroids and dimensions of grids were centered on each of the proteins’ binding sites and only the ones that reproduced the experimental ligands poses were retained as shown in Table 1 and used in the virtual screening procedure. The rmsd values from the docking validation results ranged from 0.86 to 1.88 Å with co-crystallized inhibitors of Pim-1 kinase and P38-MAPK showing the best dock poses. Since all the values are within acceptable range, they were then used to carry out the docking calculations.

**Table 1:** Validation of the docking protocol employed in the study

Target	PDB Code	Grid Box Origin			Grid Box Radius			RMSD
		X	Y	Z	X	Y	Z	
A_2_AR	2YDO	−28.9352	9.0723	−23.3486	4.0	7.0	4.0	1.60
BTK	4OTF	−37.9926	26.1210	−9.4603	7.0	6.0	10.5	1.56
CS	5QC5	42.8295	−4.4045	42.1155	8.0	8.0	8.0	1.65
Il-17α	5HI4	78.6552	−44.1998	−45.5098	8.0	8.0	8.0	1.88
Il-23	3QWR	23.5974	−26.6082	−51.9151	8.0	8.0	8.0	1.72
JaK-3	5TTS	−0.2570	17.8777	−5.2946	7.7	8.0	7.0	1.53
P38-MAPK	3NEW	24.4628	16.3186	10.6828	5.0	5.5	4.9	0.94
PDE-4	5K1I	12.5596	3.8457	68.4582	6.0	6.0	6.3	1.48
PAD	4X8G	26.8987	45.0298	26.6235	8.0	8.0	8.0	1.64
Pim-1 Kinase	4A7C	−41.1394	−3.0677	2.5015	6.0	5.5	6.2	0.86
PKC	5F9E	26.2347	78.7212	29.1280	8.0	6.0	8.0	1.16
RAC-1	5VCU	2.3215	−21.9062	−6.8930	8.0	5.0	8.0	1.23
SPK	4XG6	0.4376	−3.1674	7.0491	6.0	7.0	6.0	1.86
S1PR	3V2W	7.4851	17.6949	−8.9697	8.0	8.0	8.0	1.66
TNF-α	2AZ5	−19.0000	74.2776	33.5624	6.0	6.3	6.8	1.47

*A*_*2*_*AR* Adenosine A2 Receptor, *BTK* Bruton’s Tyrosine Kinase, *CS* Cathepsin S, *IL-17A* Interleukin-17A, *IL-23* Interleukin-23, *JaK-3* Janus Kinase 3, *P38-MAPK* Mitogen-activated Protein Kinase-p38, *PDE-4* Phosphodiesterase-4, *PAD* Peptidylarginine Deiminase, *Pim-1 kinase* ProviralIntegration site for Moloney Murine Leukemia Virus-1 Kinase, *PKC* Protein Kinase C, *RAC-1* Ras-related C3 Botulinum Toxin Substrate-1, *S1PR* Sphingosine 1-Phosphate Receptor, *SPK* Protein Kinase C, and *TNF-α* Tumor Necrosis Factor-alpha

**Fig. 1:**
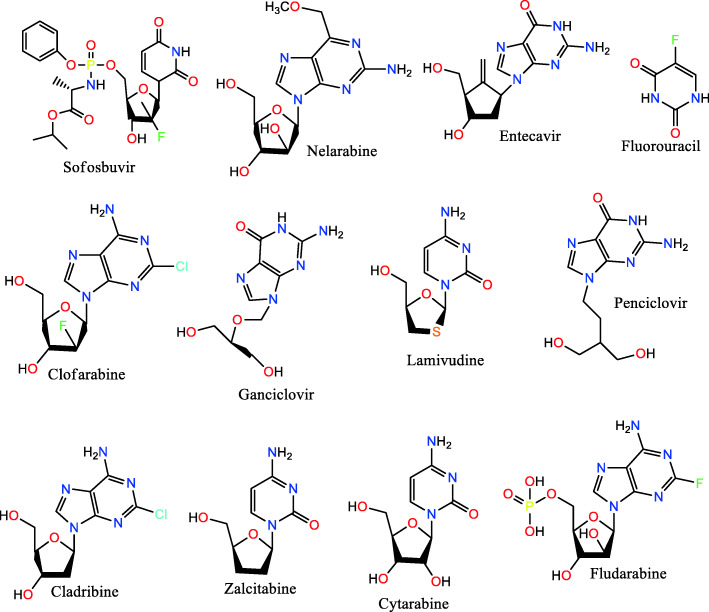
2-dimensional structures of the twelve drugs that showed higher binding affinity for all the studied 15 protein targets than each of their corresponding co-crystallized ligands

The distribution of the docking scores of the approved drugs across the studied targets ranged from low negative values (− 22.81 kcal/mol) to positive values (Table 2). The most susceptible protein to the dataset was observed to be the tyrosine kinase (JAK-3) which is confirmed to participate in the inflammatory affliction. About 35% of the dataset demonstrated higher binding affinity for JAK-3 than its specific co-crystallized inhibitor. This lends credibility to our docking method because JAK-3 had been reported to be a promiscuous protein [33]. Next to JAK-3 are IL-17A, PKC, A_2_AR and P38-MAPK respectively with about 9, 5, 4 and 4% of the dataset scoring lower than its co-crystallized ligands. The rest have a low percentage of the dataset in that category. The least interactive protein was IL-23 which has only thirteen drugs scoring better than its co-crystallized ligand. These observations are consistent with earlier reports [34]. We compared the performance of the thirteen compounds across the other 14 targets and realized all of them scored higher than all the co-crystallized ligands except one. In other words, inhibitors of IL-23 will likely exhibit broad spectra of activities against various types of psoriasis. Interestingly, all the twelve drugs are either purine or pyrimidine nucleoside/nucleotide analogues, except for fluorouracil which is a simple pyrimidine (uracil) derivative with neither phosphate group nor modified sugar side chain (Fig. 1).

**Table 2:** Number of the drugs with higher binding affinity than the co-crystallized ligands for each studied protein targets, their corresponding percentages of the dataset and the docking score of the topmost scorer

Protein Target	Co-crystallized ligand (kcal/mol)	No. of compounds with higher binding affinity than co-crystallized ligands	Percentage of dataset with higher binding affinity than co-crystallized ligands	Maximum score (kcal/mol)
A_2_AR	−12.62	79	4.27	−16.84
BTK	− 13.26	35	1.91	− 18.26
CS	−11.65	55	2.99	−17.38
IL-17A	−10.59	161	8.76	−15.64
IL-23	−11.91	13	0.73	−15.62
JAK-3	−9.73	641	34.64	−16.31
P38-MAPK	−12.32	78	4.24	−17.67
PDE-4	−13.91	36	2.06	−16.74
PAD	−13.91	24	1.33	−18.23
Pim-1 Kinase	−14.68	14	0.8	−19.08
PKC	−12.23	97	5.24	−18.92
RAC-1	−16.08	19	1.05	−20.91
SPK	−13.02	107	1.23	−16.64
SIPR	−17.36	19	1.06	−22.81
TNF-α	−10.81	46	2.5	−15.52

**Fig. 2:**
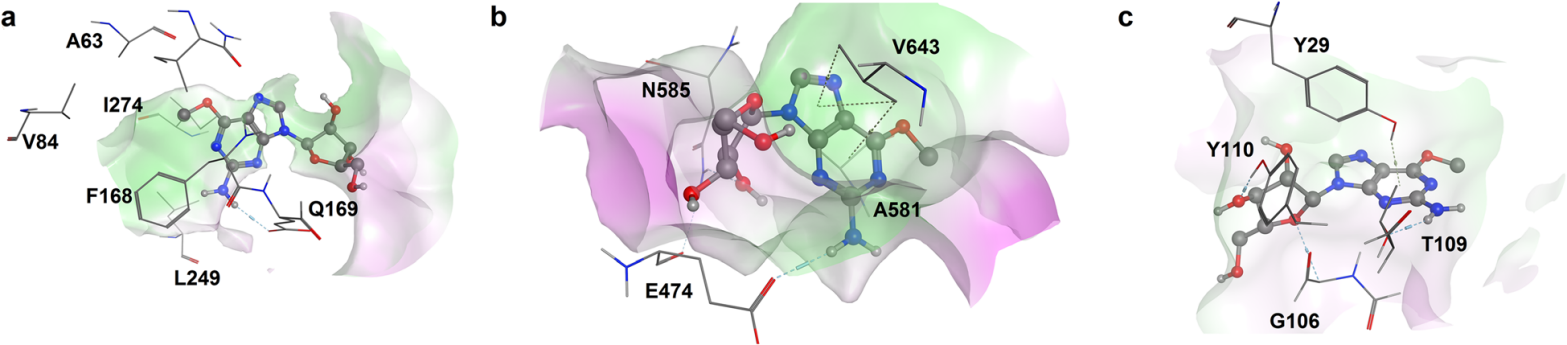
Binding poses of nelarabine within the binding cavities of **a** A_2_AR, **b** PAD and **c** SIP receptor. In each of them carbon atoms are colored grey while ligand molecules are presented in ball and stick format. Hydrogen, arene-H and arene-carbon bonds are shown in cyan and light yellow respectively. Only interacting amino acid residues are shown for clarity

The interesting binding interactions shown by the 12 compounds toward the selected protein targets could be based on their highly functionalized nature, with many polar moieties and the presence of cyclic aromatic groups whose pi-electrons are readily available to engage into hydrophobic bonding. The polar nature of the drugs is also evident from their molecular structures. Nelarabine, entecavir, clofarabine, ganciclovir, penciclovir, and fludarabine are derivatives of purine base while sofosbuvir, fluorouracil, zalcitabine, and cytarabine are derivatives of pyrimidine base. These polar moieties can easily be involved in H-bonding. Current indications of the 12 drugs presented in Table 3 show that they are either antiviral or anticancer drugs. Thus, purine and pyrimidine based antiviral and anticancer drugs are potential chemotherapeutic options for handling psoriasis. Examination of the individual scores of each of the twelve for the 15 targets (Table 4) revealed nelarabine as the topscorer for five targets (A_2_AR, PAD, SPK, SIP receptor and TNF-α), followed by fludarabine with highest binding affinity for IL-17, JAK-3 and PKC. Apart from cladribine which topped for CS and PDE-4, the rest of the drugs either emerged as the topscorer for just one target or none. Nelarabine poses in the binding sites of A_2_AR, PAD and SIP receptors as shown in Fig. 2 confirmed our earlier hypothesis as it was observed to make a series of arene-carbon, arene-H and pi-pi interactions with the various protein binding sites residues.

**Table 3:** Names, chemical classes and current indications of the 12 drugs

Drug Name	Chemical Class/ Structural Scaffold	Current Indications	References
Sofosbuvir	Pyrimidine nucleotide analog	Hepatitis C	[35]
Nelarabine	Purine nucleoside analog	Acute lymphoblastic Leukemia	[36]
Entecavir	Purine nucleoside analog	Hepatitis B	[37]
Clofarabine	Purine nucleoside analog	Acute lymphoblastic leukemia	[38]
Ganciclovir	Purine nucleoside analog	Cytomegalovirus infection	[39]
Fluorouracil	Pyrimidine analog	Various cancers	[40]
Penciclovir	Purine analog	Herpes virus infections	[41]
Lamivudine	Pyrimidine nucleoside analog	HIV/AIDS, Hepatitis B	[42]
Cladribine	Purine nucleoside analog	B-cell chronic lymphocytic leukemia	[43]
Zalcitabine	Pyrimidine nucleoside analog	HIV/AIDS	[44]
Fludarabine	Purine nucleotide analog	Leukemia and Lymphoma	[45]
Cytarabine	Pyrimidine nucleoside analog	Leukemia and Lymphoma	[46]

**Table 4:** Binding free energies of the twelve drugs across the 15 selected targets

Targets	Binding Free Energies (kcal/mol)											
	Sofosbuvir	Nelarabine	Entecavir	Clofarabine	Ganciclovir	Fluorouracil	Penciclovir	Lamivudine	Cladribine	Zalcitabine	Fludarabine	Cytarabine
A_2_AR	−13.78	−16.85	−15.88	−13.126	−15.46	− 14.41	− 13.93	− 14.24	−13.54	− 13.22	− 14.89	−13.22
BTK	− 16.24	− 18.04	−15.87	− 15.585	− 16.69	− 16.05	− 16.02	− 16.25	− 17.92	− 18.72	− 16.10	− 16.99
CS	− 13.83	− 15.32	− 12.27	− 13.800	− 13.93	− 12.70	− 12.76	− 13.32	− 17.03	− 12.80	− 13.48	− 13.90
IL-17α	−13.69	− 14.77	− 14.84	− 13.935	−14.44	−13.61	− 13.63	−14.96	−13.56	− 13.61	− 15.96	−14.47
IL-23	− 13.22	−14.94	− 12.05	− 15.623	−13.01	− 14.82	−14.24	−13.36	− 14.37	− 12.51	−15.49	−13.82
JaK-3	−13.25	− 14.81	− 13.43	− 14.571	−14.94	− 14.86	− 13.99	−13.24	−14.77	− 12.59	−15.15	− 13.51
P38MAPK	− 14.25	− 15.06	− 14.39	− 13.181	− 15.53	−13.43	−14.13	− 13.67	−14.47	−13.29	− 14.44	−14.97
PDE-4	−13.99	− 13.98	− 12.06	− 14.206	− 12.60	−12.79	− 12.49	− 13.29	−14.41	− 11.90	− 13.63	− 13.05
PAD	−13.98	−18.23	− 15.86	− 15.212	− 15.48	− 15.44	−14.84	− 15.67	− 15.62	− 15.37	−15.34	−14.89
Pim-1 Kinase	−16.99	− 16.47	− 17.29	− 16.500	− 19.08	− 17.33	− 17.48	−17.19	−14.68	−16.44	− 16.83	−16.18
PKC	−17.21	− 18.03	−16.37	− 16.529	− 17.49	− 16.66	−16.51	− 16.70	−16.80	− 16.32	− 18.15	− 17.22
RAC-1	−19.61	−18.20	− 18.88	− 19.022	− 20.14	− 20.24	− 20.91	− 18.15	−18.59	− 17.15	− 18.70	− 19.34
S1PR	−20.31	− 22.28	− 20.05	−20.333	−19.66	− 20.38	− 21.78	− 18.98	−20.00	−18.30	−21.90	− 20.27
SPK	−12.82	−15.32	− 13.63	− 12.929	− 14.66	− 13.19	− 13.98	− 11.56	−15.10	− 13.95	− 13.77	− 12.36
TNF-α	− 13.10	− 13.36	− 11.47	−12.485	− 12.24	−12.44	− 11.95	− 12.51	− 11.97	−12.41	− 12.00	−11.96

These 12 known drugs (shown in Table 3 and Fig. 2) identified by the described computational approach could be possible candidates for the treatment of psoriasis. These are essentially antiviral or antineoplastic drugs and have previously been reported to show some side effects. As an example, common side effects of sofosbuvir (administered as tablets with brand name: Sovaldi) include: fatigue, headache, nausea, insomnia, itching, anemia, weakness and rash. This drug is a nucleotide analogue inhibitor of the hepatitis C virus (HCV) NS5B polymerase used for the treatment of chronic hepatitis C (CHC) infection. It is often employed as part of a combination antiviral treatment regimen. Fluorouracil has been administered as a topical cream and as an injection. It has appeared in several brand names (including adrucil) approved by the FDA as an injection acting as a nucleoside metabolic inhibitor for the treatment of patients with several cancer types, including adenocarcinoma of the colon and rectum, adenocarcinoma of the breast, gastric adenocarcinoma and pancreatic adenocarcinoma. The drug causes hair loss, nausea, bruising, among other effects (RxList, https://www.rxlist.com/adrucil-drug.htm#description). Cytarabine is an injectable cancer medication for the treatment of certain types of blood cancers (leukemia), particularly those associated with meningitis. Common side effects include nausea and vomiting, appetite loss, diarrhea, constipation, headache, dizziness, injection site reactions (e.g. pain, swelling, and redness), drowsiness, weakness, memory problems, back pain, pain in your arms or legs, or trouble sleeping (insomnia). Since psoriasis is a skin disease, the administration route for the repurposing of some of these injectable drugs could avoid the use of injections to reduce the side effects observed by injecting patients, preferably by topical administration. In the case of sofosbuvir, side effects related to the ingestion of the tablets (e.g. fatigue, headache, nausea, etc.) could be avoided when administered topically. This, together with other drugs currently available as non-ingestible and non-injectable powders would be most suitable for repurposing as potential drugs for the treatment of psoriasis.

## Conclusion

In this study, we identified 12 FDA approved drugs (nelarabine, fludarabine, clofarabine, cladribine, zalcitabine, cytarabine, ganciclovir, penciclovir, sofosbuvir, entecavir, fluorouracil, and lamivudine) with higher binding affinity for all the 15 studied anti-psoriatic targets than their co-crystallized ligands. The similarity between the mechanisms of action of psoriasis and the diseases (cancer and viral) which the 12 drugs are currently indicated seems to confirm the *in-silico* prediction. A recent review on psoriasis and its treatment [47] suggests that none of these twelve drugs is currently prescribed for managing the psoriatic condition. Although some drugs used for managing psoriasis at the moment, like metronidazole, share a common structural motif with our virtual hits, we consider these twelve compounds as possible candidates that can be repositioned for managing psoriasis. In the future, we hope to validate the anti-psoriatic property of the 12 drugs in a biological assay.

## Data Availability

All data generated or analysed during this study are included in this published article.

## Abbreviations

3-D: Three dimensional
P38-MAPK: Mitogen-activated Protein Kinase-p38
PAD: Peptidylarginine Deiminase
PDB: Protein data bank
PDE-4: Phosphodiesterase-4
Pim-1 kinase: ProviralIntegration site for Moloney Murine Leukemia Virus-1 Kinase
PKC: Protein Kinase C
RAC-1: Ras-related C3 Botulinum Toxin Substrate-1
S1PR: Sphingosine 1-Phosphate Receptor
SPK: Protein Kinase C
TNF-α: Tumor Necrosis Factor-alpha
